# Severe CSF immune cell alterations in cryptococcal meningitis gradually resolve during antifungal therapy

**DOI:** 10.1186/s12883-024-03742-9

**Published:** 2024-07-03

**Authors:** Christine Dambietz, Michael Heming, Tobias J. Brix, Andreas Schulte-Mecklenbeck, Phil-Robin Tepasse, Catharina C. Gross, Jonel Trebicka, Heinz Wiendl, Gerd Meyer zu Hörste

**Affiliations:** 1 Department of Neurology With Institute of Translational Neurology, University Hospital Münster, Albert-Schweitzer-Campus 1, Bldg A1 48149, Münster, Germany; 2 Department of Gastroenterology, Hepatology, Endocrinology and Infectiology, University Hospital Münster, Münster, Germany; 3 Institute of Medical Informatics, University of Münster, Münster, Germany

**Keywords:** Fungal meningitis, Cryptococcus neoformans, HIV, Antifungal therapy, Intrathecal immunity, Flow cytometry

## Abstract

Cryptococcal meningitis (CM) is a severe fungal disease in immunocompromised patients affecting the central nervous system (CNS). Host response and immunological alterations in the cerebrospinal fluid (CSF) after invasion of *Cryptococcus neoformans* to the central nervous system have been investigated before but rigorous and comprehensive studies examining cellular changes in the CSF of patients with cryptococccal meningitis are still rare. We retrospectively collected CSF analysis and flow cytometry data of CSF and blood in patients with CM (*n* = *7*) and compared them to HIV positive patients without meningitis (*n* = *13*) and HIV negative healthy controls (*n* = *7*). Within the group of patients with CM we compared those with HIV infection (*n* = *3*) or other immunocompromised conditions (*n* = *4*). Flow cytometry analysis revealed an elevation of natural killer cells and natural killer T cells in the CSF and blood of HIV negative patients with CM, pointing to innate immune activation in early stages after fungal invasion. HIV positive patients with CM exhibited stronger blood-CSF-barrier disruption. Follow-up CSF analysis over up to 150 days showed heterogeneous cellular courses in CM patients with slow normalization of CSF after induction of antifungal therapy.

**Supplementary Information:**

The online version contains supplementary material available at 10.1186/s12883-024-03742-9.

## Introduction

Infectious meningitis is caused by various pathogens including bacteria, viruses, fungi or parasites. Common symptoms of meningitis are headache, fever, neck stiffness, nausea and vomiting, confusion or reduced level of consciousness and ocular symptoms including photophobia, diplopia or loss of vision [1, 2]. The most common fungal meningitis is caused by *Cryptococcus neoformans* which is a yeast that occurs ubiquitously in the natural environment. Inhalation of cryptococcal spores and desiccated yeast can lead to pulmonary colonization and latent fungal infection but is usually well controlled in immunocompetent people. Cryptococcal dissemination and clinical manifestations of cryptococcal infection develop mostly in immunocompromised patients, e.g. in later HIV stages and AIDS, after organ transplantation or in patients on other immunosuppressive treatments. Fungal pathogens can then replicate and disseminate and infect any other organ. It has been shown that *Cryptococcus neoformans* has a propensity to disseminate to the central nervous system (CNS), leading to cryptococcal meningitis (CM) or meningoencephalitis [2, 3]. Often, infection of the meninges can lead to elevated intracerebral pressure (ICP) due to cryptococcal polysaccharides blocking drainage of cerebrospinal fluid (CSF) or altered vascular permeability [4, 5]. Cryptococcal replication in the brain parenchyma is associated with formation of nodular lesions, so-called cryptococcomas [6]. Early diagnosis and therapy determine the course of CM which can be fatal [1].

Lumbar puncture (LP) and analysis of CSF is essential to diagnose meningitis. Changes in standard CSF parameters are not specific for individual pathogens in meningitis. Flow cytometry of CSF can facilitate deeper CSF cell characterization in certain diseases [6–9], hint towards involvement of various cell types in underlying pathological mechanisms and support neurological diagnoses. A previous flow cytometry analysis of the CSF was reported in patients with HIV-associated CM [7, 8] but that study aimed to detect cryptococcal pathogens rather than host CSF cells in patients with CM and did not include healthy controls or HIV patients without CM. The host cellular response of the CSF to CNS invasion of *Cryptococcus neoformans* and changes after induction of antifungal therapy remain poorly characterized.

In this retrospective study we characterized the cellular composition in blood and CSF of patients with CM and compared them to groups of immunocompromised HIV positive patients without CM and HIV negative healthy controls. This revealed partly distinct cellular alterations in patients with CM with either HIV infection (CM/HIV+) or other immunocompromised conditions (CM/HIV-). CM/HIV+ patients showed partial overlap of changes in a HIV positive control group without meningitis. Follow-up of routine parameters and cellular reconstitution kinetics showed slow normalization of standard CSF parameters and immune cell alterations after initiation of antifungal therapy.

## Methods

### Patients

At the University Hospital Münster, CSF is obtained from patients presenting with neurological or psychiatric symptoms during diagnostic work-up. All CSF samples are analyzed in a specialized laboratory. This includes CSF standard analysis and a standardized flow cytometry panel (see below). We screened the database of the University Hospital Münster for patients that were diagnosed with CM or HIV based on the ICD-10 diagnosis and had received standard and flow cytometry analysis of CSF. All patients presented to a department of the University Hospital Münster with neurological symptoms or cognitive impairment and thus underwent LP during diagnostic workup (Table 1).

**Table 1:** Patient demographics. Demographic information on CM patients and control groups including sex, age, comorbidities, HIV stage and results from routine and flow cytometry analysis from CSF and blood.

Group	ID	Sex	Age (y)	HIV	CDC	Comorbidities	LP day 0	Bc	cMono	iMono	ncMono	CD4	CD4CD8	HLADR CD4CD8	HLADR CD4	CD45	CD8	HLADR CD8
CM	02	m	55.8	pos	C3	HIV	CSF d0	2.63	72.54	4.15	22.28	2.8	0.34	50	44.9	12.22	95.78	87.99
							Blood d0	10.24	63.3	17.78	19.17	28.64	1.19	85.71	66.86	48.07	62.71	80.81
CM	06	f	56.5	neg		condition after small bowel transplant (04/2011)	CSF d0	2.28	46.89	4.8	49.97	74.6	0.63	37.14	35.94	94.2	23.04	25.78
							Blood d0	38.27	82.74	7.63	9.67	74.7	0.72	33.33	48.71	86.47	20.24	60.71
CM	10	m	36.2	pos	C3	HIV	CSF d0	4.55	57.7	6.03	38.22	13.02	3.22	37.25	39.32	13.25	80.97	27.09
							Blood d0	11.72	80.14	1.26	19.55	9.34	5.42	2.2	2.02	94.46	83.92	2.49
CM	12	f	76.1	neg		B cell non-Hodgkin's lymphoma	CSF d0	0.08	3.18	94.5	2.23	67.34	1.59	38.57	36.29	90.54	30.37	54.17
							Blood d0	0.04	31.66	29.13	40.73	42.75	1.12	16.09	18.61	76.93	54.7	14.58
CM	21	m	46.1	pos	C3	HIV	CSF d0	3.46	92.52	0	8.11	12.01	4.85	3.68	2.06	87.03	81.48	7.13
							Blood d0	11.27	48.69	14.39	39.01	25.68	1.51	6.35	1.7	82.08	70.3	16.15
CM	22	m	77.6	neg		condition after kidney transplant (03/2019)	CSF d0	61.27	83.02	5.92	12.24	52.35	4.6	91.52	61.1	24.47	41.01	94.9
							Blood d0	NA	NA	NA	NA	NA	NA	NA	NA	NA	NA	NA
CM	24	m	43.3	neg		Sarkoidosis, condition after cryptococcoma resection (2009)	CSF d0	0.19	80.43	0	20.65	67.42	5.66	8	6.38	3.05	26.47	20.51
							Blood d0	23.13	68.05	5.08	28.43	34.01	3.16	5.64	12.38	70.57	56.47	12.49
HIV	01	m	41.3	pos	A2	condition after Syphilis	CSF d0	1.11	51.17	0	50.7	56.25	0.58	20	23.46	15.18	41.9	54.7
							Blood d0	10.39	57.75	12.15	31.93	61.83	1.24	8.5	7.17	51.09	34.24	16.82
HIV	05	f	33.6	pos	B3	HIV-associated encephalopathy	CSF d0	8.34	88.42	0	13.13	32.31	1.4	42.86	32.3	14.68	63.68	72.69
							Blood d0	9.78	89.99	1.65	8.36	36.12	2.32	25.13	14.51	73.26	59.04	29.45
HIV	07	m	42.5	pos	C3	PML (03/2011)	CSF d0	1.14	35.89	4.31	60.77	21.88	3.76	30.41	13.47	19.37	73.53	31.41
							Blood d0	2.34	81.71	2.34	17.38	11.8	2.06	24.87	16.73	82.3	85.49	24.16
HIV	08	m	48.9	pos	C3	HIV-associated polyneuropathy	CSF d0	2.01	17.47	0.03	83.02	34.41	2.55	5.41	10.2	14.14	62.01	20.2
							Blood d0	21.52	84.65	1.4	14.82	61.9	1.75	26.98	23.25	94.08	29.37	17
HIV	11	m	50.2	pos	A2	previous Syphilis with CNS manifestation	CSF d0	73.63	38.08	0	61.92	70	0.57	33.33	23.45	12.78	27.92	63.51
							Blood d0	8.16	88.57	2.51	9.15	78.22	1.15	47.13	10.76	90.3	15.76	10.34
HIV	15	m	41.5	pos	n.a	first diagnosis of HIV, neuralgic shoulder amyotrophy	CSF d0	1.12	61.41	0.54	39.13	20.99	1.13	66	34.48	58.86	76.08	78.69
							Blood d0	7.33	70.65	10.18	19.9	36.43	1.1	26.71	11.65	95.91	60.43	35.25
HIV	17	m	54.9	pos	C2	first diagnosis of HIV, Syphilis (stage II), CMV retinitis	CSF d0	19.16	79.58	2.04	19.24	41.89	1.88	50.75	31.67	76.1	43.55	68.39
							Blood d0	25.57	68.51	7.04	25.15	60.85	2.69	18.33	19.31	82.27	30.86	24.95
HIV	18	m	72.3	pos	B3	Pneumocystis jiroveci pneumonia, syringomyelia	CSF d0	11.08	42.39	0	58.7	25	0.96	0	25	6.73	71.63	34.23
							Blood d0	7.88	77.72	4.12	20.61	78.2	2.2	40.3	20.73	79.46	15.78	40.21
HIV	20	m	47.1	pos	C3	PML (2001, JCV in CSF positive)	CSF d0	0.15	73.39	0	27.47	78.21	2.42	13.33	12.59	27.21	18.89	40.6
							Blood d0	9.98	85.24	3.17	12.04	84.27	1.29	11.19	6.48	84.54	11.8	6.44
HIV	23	f	41.3	pos	B3	HIV-associated encephalitis	CSF d0	2.42	88.03	0	13.25	14.06	0.63	46.07	27.57	72.11	83.99	85.03
							Blood d0	21.11	69.05	15.44	15.35	9.84	0.39	71.19	17.83	90.2	85.19	55.48
HIV	25	m	47.9	pos	n.a	first diagnosis of HIV, HIV-associated meningitis	CSF d0	1.54	59.24	0.59	41.98	42.19	1.59	33.45	9.34	68.5	54.88	81.22
							Blood d0	13.29	81.46	3.74	16.19	77.97	1.1	16.48	6.44	82.33	18.84	33.54
HIV	26	m	41.8	pos	C1	HIV-associated neurocognitive deficiency, myelopathy, spinal disc herniation L5/S1	CSF d0	0.68	55.24	0	45.71	8.5	0.25	50	52.24	8.16	89.34	58.66
							Blood d0	7.24	89.26	1.65	9.57	31.64	2.04	18.85	33.36	86.48	59.75	19.82
HIV	27	m	69.6	pos	C3	HIV-associated neurocognitive deficiency, polyneuropathy, Brachial plexus injury	CSF d0	1.22	44.78	0	55.6	51.5	2.11	38.46	17.51	23.47	44.11	66.67
							Blood d0	9.67	92.77	0.03	7.74	44.46	0.93	31.03	6.87	90.12	51.65	11.88
Ctrl	03	m	55.4	neg		Somatic symptom disorder, depression	CSF d0	0.33	90.91	0	9.09	43.32	0.36	0	9.17	3.18	53.79	32.89
							Blood d0	19.99	81.62	4.36	14.64	50.72	1.46	5.93	6.78	11.78	37.74	6.74
Ctrl	04	m	37.8	neg		Tension headache	CSF d0	0.56	31.45	0.46	70.05	77.64	1.33	31.03	14.19	10.2	20.21	28.05
							Blood d0	8.43	91.19	3.29	6.37	63.99	0.76	18.68	8.8	67.44	30.6	15.9
Ctrl	09	f	71.7	neg		NA	CSF d0	2.74	69.49	0.56	28.81	77.84	1.77	31.25	20.57	12.77	18.95	59.65
							Blood d0	12.24	50.67	7.03	45.55	90.63	0.73	11.66	3.98	87.54	7.27	11.22
Ctrl	13	m	37.4	neg		dysestheia of unknown etiology	CSF d0	1.61	32.03	0.34	68.08	77.05	2.33	33.33	34.21	14.37	17.83	66.96
							Blood d0	10.43	80.38	6.31	13.91	73.68	1.37	9.79	8.45	76.52	15.09	23.4
Ctrl	14	m	68.6	neg		Transient global amnesia	CSF d0	3.79	46.32	0	53.68	56.8	0	NA	15.49	3.17	40.8	47.06
							Blood d0	10.64	79.49	8.2	12.81	84.83	0.31	20	8.12	87.7	12.38	14.55
Ctrl	16	f	56.1	neg		Neuralgiform headache	CSF d0	2.84	80.12	0	20.47	83.86	2.57	16	10.42	19.02	13.05	46.46
							Blood d0	18.57	89.26	2.65	8.08	90.72	0.89	9.41	7.47	50.02	5.15	6.29
Ctrl	19	m	46.8	neg		progressive muscle weakness of unknown etiology	CSF d0	0.66	59.52	0.34	41.5	66.78	1.55	28.13	10.43	18.68	30.75	22.48
							Blood d0	13.32	79.53	6.14	14.67	68.84	1.92	7.78	8.67	86.73	26.29	9.3

Group	Granulo	Lympho	Mono	NK	NKT	Plasma	Tc	HLADR Tc	CD4CD8ratio	dimNK	brightNK	Cells/µl	Lympho/µl	Granulo/µl	Ery/µl	Protein	Albumin CSF	Albumin serum
CM	13.89	79.62	4.2	0.11	0.79	0.05	95.82	86.5	0.0292337	25	25	0	0	0	4300	774	201	
	95.96	2.13	1.65	5.07	2.05	0	57.56	77.29	0.4567055	69.23	3.85							19.9
CM	63.73	33.19	1.97	34.54	6.98	0.36	16.75	33.46	3.2378472	8.28	85.9	81	48	33	0	2300	1150	
	88.83	2.65	8.36	24.12	4.06	0.22	15.05	52.53	3.6907115	59.25	31.88							37.1
CM	23.6	49.75	24.39	5.55	1.82	0	71.94	28.89	0.1608003	12.3	72.92	NA	NA	NA	NA	NA	NA	
	26.96	38.93	34.05	4.92	5.81	0	73.25	2.52	0.1112965	61.49	34.27							NA
CM	9	77.65	11.36	5.89	1.8	0	87.46	41.74	2.2173197	41.78	54.41	100	71	21	0	1570	767	
	38	47.34	13.83	10.23	18.33	0.01	69.23	16.23	0.7815356	94.07	4.74							35.3
CM	5.21	48.43	42.22	7.65	1.04	1.16	75.76	6.37	0.1473981	22.99	6.47	39	39	0	0	911	214	
	64.57	20.27	14.99	8.05	2.96	0.99	69.77	12.24	0.3652916	86.23	10.94							28.9
CM	11.81	76.38	9.81	46.38	13.04	0.05	36.4	76.65	1.2765179	0.59	99.32	0	0	0	0	993	542	
	NA	NA	NA	NA	NA	NA	NA	NA	NA	NA	NA							NA
CM	12.13	63.02	21.67	4.86	11.21	0	82.62	10.18	2.5470344	7.69	88.46	0	0	0	0	397	221	
	63.55	18.04	18.07	13.28	22.47	0.01	33.09	12.89	0.6022667	84.46	12.61							41.5
HIV	5.49	75.15	17.71	0.88	1.11	0	95.58	36.92	0.0264916	0	75	1	1	0	0	760	424	
	58.86	32.13	8.74	24.91	2.5	0.08	56.71	11.2	1.8057827	98.12	1.49							41.5
HIV	9.87	77.13	11.57	1.16	2.55	0	86.57	59.06	0.1309673	5	70	1	1	0	0	642	344	
	65.72	29.62	3.97	5.7	1.3	0.04	75.95	24.14	0.6117886	89.73	8.73							42.9
HIV	5.66	90.91	3.04	1.36	2.56	0	92.33	27.39	0.2975656	38.82	43.53	1	1	0	0	1260	683	
	48.26	44.13	7.35	8.48	0.93	0	79.76	23.31	0.1380278	83.55	6.6							41.3
HIV	7.97	28.94	58.84	1.88	2.89	0	91.21	16.38	0.5549105	20	56.66	5	3	0	180	556	293	
	89.35	6.71	3.79	10.86	1.82	0.19	57.11	21.35	2.1075928	74.93	19.97							42.7
HIV	8.51	64.95	25.74	0.61	17.99	0	80.79	35.66	2.5071633	0	100	2	2	0	5	630	350	
	56.89	34.21	8.58	6.14	6.83	0.09	73.35	11.94	4.963198	35.6	58.14							41.1
HIV	1.95	95.06	1.89	1.24	0.51	0.4	95.83	68.85	0.2758938	71.3	8.7	7	6	0	0	656	287	
	76.88	18.96	3.87	11.46	1.63	0.09	72.97	26.42	0.6028463	95.3	1.68							45.8
HIV	4.99	89.48	2.99	1.48	0.66	0.36	78.07	47.27	0.9618829	31.41	53.93	50	43	0	0	991	358	
	78.57	9.43	11.88	8.77	3.89	0.3	57.61	21.69	1.9718082	88.24	3.82							34.2
HIV	26.94	27.31	20.37	6.49	10.54	0	56.22	31.73	0.3490158	29.17	12.5	1	1	0	0	486	338	
	85.39	7.23	7.13	26.49	5.6	0.19	51.28	24.76	4.9556401	94.97	1.27							24
HIV	6.47	78.71	14.1	1.77	2.08	0	95.23	18	4.1402859	26.09	60.87	3	3	0	0	578	346	
	69.75	19.72	10.21	18.06	2.69	0.13	66.32	6.85	7.1415254	79.67	18.47							48.2
HIV	1.56	96.19	1.5	0.44	0.09	0.75	93.82	76.42	0.1674009	33.33	7.58	42	40	0	9	505	198	
	31.56	62.38	5.54	2.61	0.23	0.28	75.21	51.51	0.1155065	90.95	0.28							41.6
HIV	6.01	83.81	9.06	0.59	0.29	0.86	95.33	49.43	0.7687682	37.5	36.61	12	12	0	0	1140	580	
	61.83	29.78	6.56	10.72	2.39	0.5	67.77	12.21	4.138535	96.19	1.37							38.6
HIV	8.17	72.36	17.33	0.91	7.3	0.11	89.85	58.12	0.0951422	25	50	0	0	0	0	521	227	
	56.49	36.66	6.69	3.38	2.92	0.17	83.27	24.56	0.5295397	64.41	12.92							41.1
HIV	25.6	58.39	11.95	1.53	2.52	0	94.04	40.86	1.1675357	45	50	0	0	0	0	668	331	
	83.69	12.59	3.54	18.61	6.29	0.01	55.12	9.83	0.8607938	11.22	0.38							39.8
Ctrl	47.46	36.71	14.61	2.96	2.63	0	91.12	23.1	0.8053542	22.22	66.67	1	1	0	0	347	194	
	45.05	48.69	5.73	14.43	5.9	0.07	56.43	7.56	1.3439322	93.91	2.79							43.8
Ctrl	13.15	61.42	23.16	1.22	2.35	0	95	17.51	3.8416625	3.57	60.71	0	0	0	0	539	263	
	69.95	24.69	5.26	10.59	4.1	0.17	69.55	11.88	2.0911765	83.88	3.92							48.6
Ctrl	10.69	80.3	7.22	2.84	1.68	0	91.67	28.42	4.1076517	8.93	55.36	NA	NA	NA	NA	NA	NA	
	46.82	36.01	6.77	12.64	1.41	0.12	70.38	4.83	12.4663	94.78	1.81							NA
Ctrl	12.07	38.18	48.69	1.9	1.76	0	94.44	41.09	4.3213685	15.38	46.15	1	1	0	0	456	222	
	69.76	23.26	6.59	21.71	5.93	0.03	58.85	12.51	4.8827038	93.35	2.07							48.7
Ctrl	16.01	46.98	33.81	3.03	0.76	0	94.7	28.8	1.3921569	25	50	1	1	0	0	459	248	
	73.02	17.06	9.69	23.18	2.57	0.03	52.6	9.42	6.8521809	89.81	0.6							41.1
Ctrl	5.27	78.6	12.7	1.42	0.47	0	91.97	15.52	6.4260536	26.67	0	0	0	0	0	314	186	
	70.35	17.47	11.78	8.54	3.08	0.21	58.56	7.83	17.615534	93.06	0.86							41.9
Ctrl	2.85	84.36	11.64	0.89	1.03	0	97.04	14.65	2.1717073	36.84	36.84	1	1	0	0	300	164	
	51.85	35.28	12.7	23.72	2.19	0.11	56.84	9.05	2.6184861	94.89	2.15							39.6

Group	Albumin ratio	IgG CSF	IgGSerum	IgG ratio	IgA CSF	IgA Serum	IgA ratio	IgM CSF	IgM Serum	IgM ratio	Glucose ratio	Lactate	Ig synthesis	IgA synthesis	IgM synthesis	BCBD	OCB
CM	10.1	213		16.5	6.85		6.1	20.7		6.3	0.35	2.07	52	0	54	1	pos
			12.9			1.13			3.3								
CM	31	174		17.5	42.4		18.2	8.66		8	0.3	5.88	0	0	0	1	neg
			9.93			2.33			1.08								
CM	NA	NA		NA	NA		NA	NA		NA	NA	NA	NA	NA	NA	NA	NA
			NA			NA			NA								
CM	21.7	59.8		10.5	11.6		8.7	2.81		5.4	0.3	4.18	0	0	0	1	neg
			5.67			1.34			0.52								
CM	7.4	228		9.5	34.1		8.2	16.4		10.3	0.5	2.21	41.5	55.1	82.9	1	pos
			24			4.14			1.59								
CM	NA	36.1		NA	6.74		NA	4.42		NA	NA	6.96	NA	NA	NA	NA	NA
			NA			NA			NA								
CM	5.3	28.2		2.6	1.42		1.3	0.1		0.2	0.5	1.88	0	0	0	0	neg
			10.5			1.08			0.68								
HIV	10.2	39.8		4.6	6.07		3.1	0.39		0.5	0.9	1.53	0	0	0	1	neg
			8.69			1.93			0.77								
HIV	8	104		5.3	9.23		2.8	1.36		1.2	0.44	1.22	0	0	0	1	neg
			19.8			3.29			1.16								
HIV	16.5	103		12.8	7.06		6.3	9.47		17.9	0.5	1.42	0	0	65.4	1	pos
			8.02			1.12			0.52								
HIV	6.9	32.7		3.6	3.12		2.2	0.1		0.4	0.75	2.34	0	0	0	0	neg
			9.19			1.44			0.369								
HIV	8.5	49.8		4.5	7.85		2.4	0.371		0.3	0.77	1.43	0	0	0	1	neg
			11.1			3.21			1.1								
HIV	6.3	63.8		4.4	5.4		1.6	0.24		0.3	0.56	1.79	0	0	0	0	pos
			14.5			3.44			0.698								
HIV	10.5	235		11.7	26.5		3.8	20.5		6.1	0.47	1.86	29	0	50	1	pos
			20.1			6.98			3.36								
HIV	14.1	43.7		6.3	16.4		5.6	0.44		1	0.4	1.44	0	0	0	1	pos
			6.91			2.92			0.42								
HIV	7.2	37.3		3.6	7.4		1.7	0.1		0.4	0.66	1.41	0	0	0	1	pos
			10.3			4.47			0.42								
HIV	4.8	81.9		4.4	4.12		2.3	2.51		1.2	0.61	1.91	26	10	26	1	neg
			18.6			1.77			2.07								
HIV	15	156		13.3	23.2		6.4	1.21		1.6	0.7	1.46	0	0	0	1	pos
			11.7			3.62			0.74								
HIV	5.5	51.5		3.5	8		1.5	0.38		0.2	0.5	1.73	0	0	0	0	pos
			14.7			5.51			1.63								
HIV	8.3	50.1		5.1	3.79		1.7	1.78		0.2	0.62	2.9	0	0	0	0	pos
			9.76			2.28			7.46								
Ctrl	4.4	21.6		1.9	3.08		1	< 0,15		< 0,1	0.4	1.77	0	0	0	0	neg
			10.9			3.08			0.85								
Ctrl	5.4	26.7		3	1.81		1.5	0.42		0.3	0.7	1.78	0	0	0	0	pos
			8.98			1.21			1.6								
Ctrl	NA	NA		NA	NA		NA	NA		NA	NA	NA	NA	NA	NA	NA	NA
			NA			NA			NA								
Ctrl	4.6	27.6		2.1	2.51		1	0.163		0.1	0.48	1.55	0	0	0	0	neg
			13.2			2.6			2.1								
Ctrl	6	30		2.7	3.77		1.4	0.508		0.4	0.58	1.71	0	0	0	0	neg
			11.2			2.65			1.35								
Ctrl	4.4	19.1		2	3.4		1	< 0,149		< 0,1	0.48	1.75	0	0	0	0	neg
			9.45			3.26			1.42								
Ctrl	4.1	22.1		1.9	0.838		0.7	< 0,156		< 0,2	0.66	1.73	0	0	0	0	neg
			11.7			1.16			0.757								

*Abbreviations: B* Blood, *Bc B* cells, *BCBD *Blood-CSF-barrier disruption, *brightNK *CD56bright natural killer cells, *CDC *Classification System for HIV Infection is the medical classification system used by the United States Centers for Disease Control and Prevention, *CM *Cryptococcal meningitis, *cMono* Classical monocytes, *Cr-Ag* Cryptococcal antigen, *CSF* Cerebrospinal fluid, *Ctrl *Healthy control group, *d* Days, *dimNK *CD56 dim natural killer cells,* Ery *Erythrocytes,* f* Female, *Granulo* Granulocytes,* HIV *Human Immunodeficiency Virus,* IgA *Immunoglobulin A,* IgG* Immunoglobulin G, *IgM* Immunoglobulin M,* iMono* Intermediate monocytes, *LP *Lumbar punctuare, *Lympho* Lymphocytes, *m *Male, *Mono* Monocytes, *NA* Not available, *ncMono* Non-classical monocytes, *neg* Negative, *NK* Natural killer cells, *NKT* Natural killer T cells, *OCB* Oligoclonal bands,* Plasma* Plasma cells, *PML* Progressive multifocal leucencephalopathy,* pos* Positive, *Tc* T cells, *y* Years

We identified seven patients with cryptococcal meningitis, for whom flow cytometry (mFC) data were available, and included them in our retrospective study, referred to as the CM group (CM; ICD-10 B45.1; *n* = *7*). Three of these patients were HIV positive (CM/HIV+ , *n* = 3) whereas the other four patients had other immunosuppressive conditions (CM/HIV-, *n* = 4) (Table 1). We further identified a control group of thirteen patients with a diagnosis of HIV without meningitis and available mFC data (HIV; ICD-10 U60; *n* = *13*). Due to HIV which is accompanied by a loss of CD4 positive T lymphocytes, those patients represent an immunocompromised control group. For further comparison, another group of seven HIV negative healthy controls was identified. Patients of both control groups presented with neurological symptoms and thus underwent lumbar puncture to establish a differential diagnosis. CSF results were categorized as normal, referring to our center’s standard values and patients of this group were diagnosed with headache, transient global amnesia or unspecific neurological symptoms of unknown etiology (Ctrl; *n* = *7*) (Table 1). All patients were admitted in one of the clinical departments of the University Hospital Münster during 2006 and 2022.

### Diagnostic workup of CM

#### Cryptococcal antigen testing, cryptococcal cultivation and Indian ink staining

With reasonable suspicion of an opportunistic infection of the CNS, a cryptococcal antigen test (Cr-Ag Lateral Flow Assay, IMMY) was performed on CSF and serum by immunochromatography with cryptococcal capsule polysaccharide as target antigen. Additional cultivation of *Cryptococcus neoformans* on Sabouraud-Agar is performed at the department of microbiology of the University Hospital Münster to verify cryptococcal infection. In the past, Indian ink staining was performed on fresh CSF to directly detect encapsulated cryptococcus through microscopy. Diagnosis of cryptococcal meningitis in our CM cohort was confirmed by either microscopy of CSF, positive cultures from CSF or serum or positivity in the cryptococcal antigen lateral flow assay of CSF and serum. Details on each patient are listed in Supplementary Table 1.

### Standard CSF analysis

CSF analysis was performed at the specialized CSF laboratory of the University Hospital Münster within one hour after LP. CSF samples were processed as described in previous works [9, 10]. CSF cells were counted in a Fuchs-Rosenthal chamber. Concentration of protein, albumin and immunoglobulins were measured by nephelometry (BN ProSpec, Siemens). ​​Evaluation of serum/CSF immunoglobulin levels for immunoglobulin A (IgA), immunoglobulin G (IgG) and immunoglobulin M (IgM) were performed by creation of a Reiber scheme. Dysfunction of the barrier between blood and CSF is referred to as blood-CSF-barrier disruption (BCBD) and was evaluated in reference to the serum/CSF albumin and IgG ratio. Oligoclonal bands (OCB) were investigated via isoelectric focusing and silver nitrate staining and were evaluated positive for intrathecal IgG synthesis.

### Flow cytometry

CSF samples and additional blood samples that are taken within regular working hours are analyzed on a standardized panel at the specialized CSF laboratory of the University Hospital Münster. All samples underwent centrifugation. Supernatant was removed and the centrifuged cell deposit was stained using antibodies against immunophenotypic surface molecules of immune cells. Staining was performed against CD3, CD4, CD8, CD14, CD16, CD19, CD45, CD56, CD138 and HLA-DR as described previously [9]. Flow cytometry was performed using a Navios flow cytometer (Beckman Coulter) and data were analyzed using the software *Kaluza* (version 2.1, Beckman Coulter). First gating was performed on forward scatter channel (FSC) and sideward scatter channel (SSC) characteristics. Further gating of cellular subsets was performed according to their specific expression profiles of clusters of differentiation as described in preceding works [10].

### Data analysis

We used *R* v4.3 to analyze the results. Statistical significance was calculated with the Mann–Whitney U-test when comparing two groups or with the Kruskal–Wallis-test and the Dunn-test as a post hoc test when comparing multiple groups. We performed multiple hypothesis testing by Benjamini-Hochberg’s procedure and included all tests in this correction (CSF: 114 tests; blood 78 tests). An adjusted *p value* < *0.05* was assessed as statistically significant. We generated heatmaps with the R package *pheatmap v1.0*. In a first step all parameters including Immunoglobulin G, Immunoglobulin A, Immunoglobulin M, Albumin, Protein, Albumin ratio, IgG ratio, IgA ratio, IgM ratio, Glucose ratio, Lactate, blood-CSF-barrier disruption (BCBD), oligoclonal bands (OCB), B cells (Bc), classical monocytes (cMono), intermediate monocytes (iMono), nonclassical monocytes (ncMono), CD4 positive T cells (CD4), CD4 and CD8 positive T cells (CD4CD8), activated CD4 and CD8 positive T cells (HLA-DR CD4CD8), activated CD4 positive T cells (HLA-DR CD4), CD8 positive T cells (CD8), activated CD8 positive T cells (HLA-DR CD8), granulocytes (Granulo), lymphocytes (Lympho), monocytes (Mono), natural killer cells (NK), natural killer T cells (NKT), Plasma cells, T cells (Tc), activated T cells (HLA-DR Tc), CD4CD8 ratio, CD56 bright natural killer cells (brightNK), and CD56 dim natural killer cells (dimNK) were normalized by the ordered quantile technique of the bestNormalize package v1.9 followed by scaling and centering [11]. Subsequently, the mean of these normalized values was calculated and visualized in a heatmap. The results were clustered by complete linkage and Euclidean distance measures. Principal component analysis (PCA) was conducted and visualized with the R package *FactorMineR v2.8* [12]. Receiver operating characteristics (ROC) analysis was performed with the *R* package *pROC v1.18* [13]. The line plots were generated with *ggplot* v3.4 with the lm method of geom_smooth to plot a linear regression line. Correlation analysis was calculated with Pearson’s correlation coefficient and p values were adjusted by Benjamini-Hochberg’s procedure.

## Results

### Identifying a cohort of patients with CM and control groups

We aimed to characterize CSF cells in CM. In total, we identified and collected data from i) seven patients with CM, ii) thirteen HIV positive patients without CM as an immunocompromised group (HIV), and iii) seven HIV negative healthy controls (Ctrl) that were age- and sex-matched to the CM group.

All CM patients had likely developed disease due to immunosuppression. Three patients were HIV positive (CM/HIV+ group, 42.8%) with one of them being on antiretroviral therapy (ART, 33%). Four CM patients were on immunosuppressive treatment but HIV- (CM/HIV- group) including corticosteroid therapy, immunosuppression after organ transplant or chemotherapy (Table 1). Two of the patients were organ transplant recipients (kidney transplant, small intestine transplant) in their medical history and they were on immunosuppressive medication (belatacept and everolimus, Ciclosporin A and Mycophenolate mofetil). One patient had steroid treatment due to sarcoidosis (ICD-10 D86) and the other one had a history of chemotherapy due to a B cell Non-Hodgkin-lymphoma (Table 1). In the CM group, the female-to-male ratio was 1:2.5 and the average age at presentation was 55.9 years. Patients of the control groups underwent LP due to neurological symptoms including headache, cognitive decline, visual disturbance, vertigo and peripheral sensory or motor symptoms. Within the HIV control group, nine patients were on antiretroviral therapy (ART, 69.2%). The female-to-male ratio was 1:5.5 and the average age was 48.7 years. We then collected data from seven matched controls with normal CSF parameters. The female-to-male ratio was 1:2.5 and the average age was 53.4 years. All groups were thus sufficiently matched in terms of sex and age.

### CM and HIV patients show severe blood-CSF-barrier disruption

**Fig. 1:**
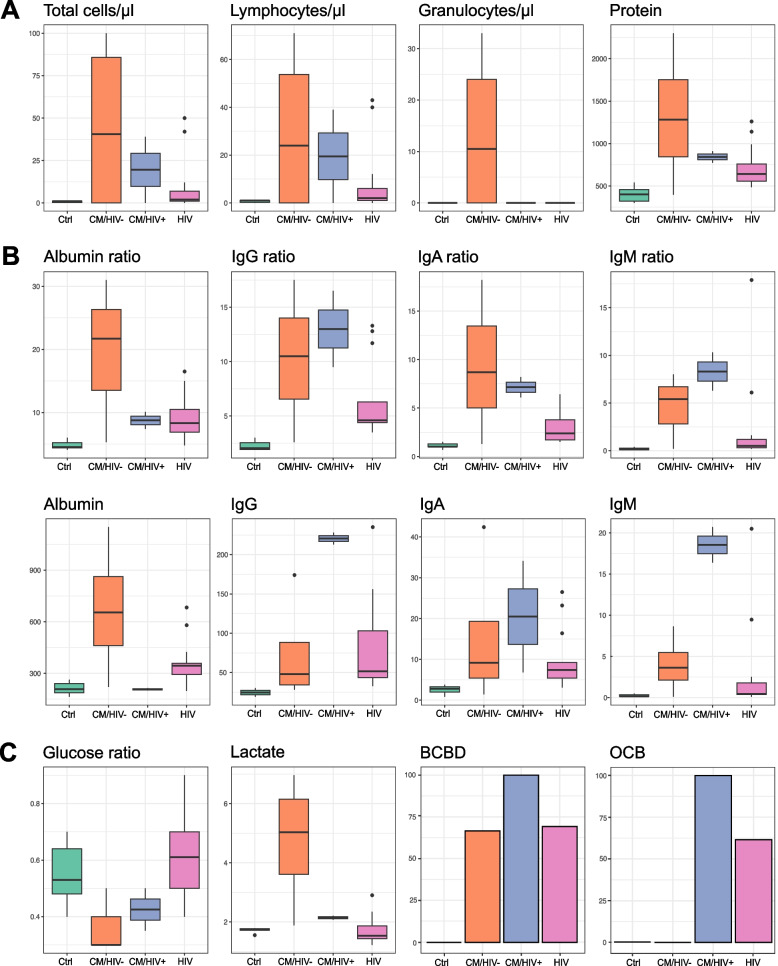
Standard CSF analysis. Basic CSF parameters indicate blood-CSF-barrier disruption and distinguish CM patients from HIV and Ctrl. **A** Cells were counted in a Fuchs-Rosenthal chamber. Protein level was measured by nephelometry. **B** Levels of albumin and intrathecal immunoglobulins were measured by nephelometry. Albumin and immunoglobulin ratios were evaluated by serum/CSF ratio.** C** Glucose ratio refers to serum/CSF levels of glucose. BCBD refers to serum/CSF ratio of albumin and immunoglobulin ratios. Oligoclonal bands were detected through isoelectric focusing and silver nitrate staining. Abbreviations - BCBD: blood-CSF-barrier disruption, CM: cryptococcal meningitis, CSF: cerebrospinal fluid, Ctrl: healthy control group, HIV: human immunodeficiency virus positive, immunocompromised control group IgA: immunoglobulin A, IgG: immunoglobulin G, IgM: immunoglobulin M, OCB: oligoclonal bands

CM is known to increase cell counts with predominance of lymphocytes and protein level in CSF and reduced CSF/serum glucose ratio and elevated opening pressure [4, 5]. First, we analyzed routine parameters of CSF. Basic CSF analysis revealed an increase of protein level (Fig. 1A), albumin (Fig. 1B) and lactate (Fig. 1C) in patients with CM. CM groups exhibited excessive blood-CSF-barrier disruption (Fig. 1C) and intrathecal immunoglobulin synthesis over all immunoglobulin subtypes with dominance of IgG (Fig. 1B) and this was higher in CM/HIV+ compared to CM/HIV- patients. *Cryptococcus neoformans* was detected in all patients with CM by Cr-Ag Lateral Flow Assay with positivity for cryptococcal antigen in CSF or serum, positive culture from CSF or serum or direct microscopy of CSF after Indian ink staining (Supplementary Table 1).

The HIV group exhibited CSF changes that partially resembled those of the CM group albeit to a lesser extent. Glucose and lactate levels were normal in HIV as expected (Fig. 1C). CSF protein, intrathecal immunoglobulins and BCBD were increased in HIV compared to control patients but to a lower extent than in the CM/HIV+ group (Fig. 1A, 1B, 1C). HIV positive patients, both with and without cryptococcal meningitis revealed positive oligoclonal bands (Fig. 1C). These changes in standard parameters of CSF were as expected in meningitis but are not specific for fungal meningitis.

### Severe changes in CSF cells in CM

**Fig. 2:**
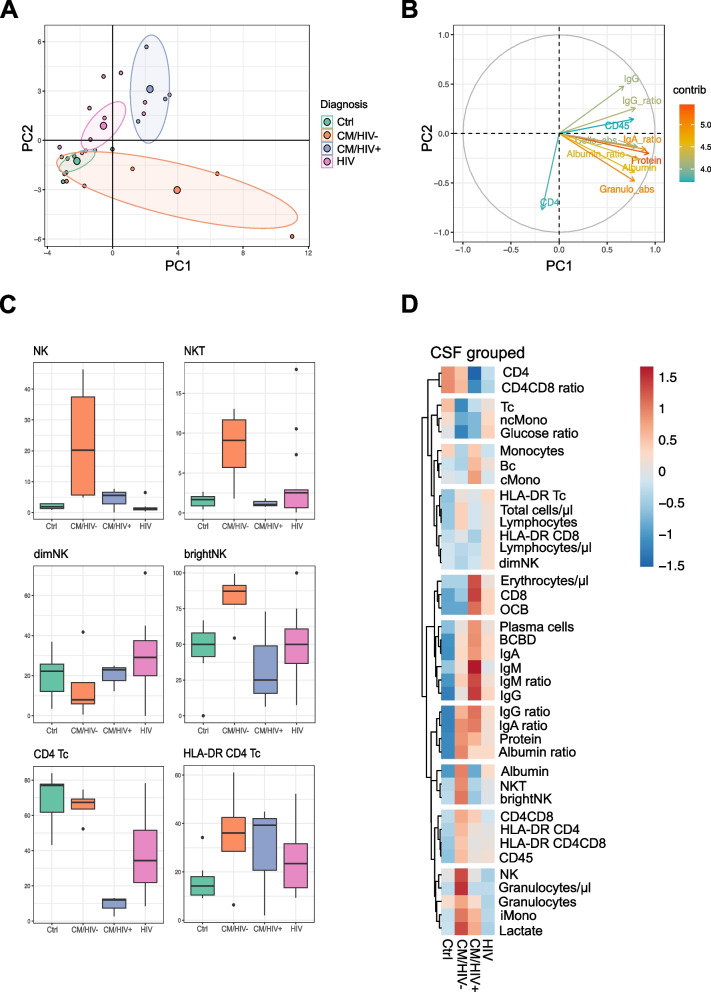
Flow cytometry analysis of CSF. CM patients show an increase of NK cells in CSF. **A** Principal component analysis of CSF. Each patient is represented as a multidimensional data point. Large circles represent the means of each group (CM/HIV+ blue, CM/HIV- orange, HIV pink, Ctrl green). Ellipses of the same color represent the confidence intervals of each group. **B** Principal components (PCs) comprise multidimensional parameters.  Parameters with the highest distinctive power determine PC1 and PC2 and are represented by the length of their vector. **C** Plotting of individual parameters revealed a significant increase of NKT and NK cells with dominance of CD56^bright^ NK cells in the CSF of the CM/HIV- group. CM patients showed elevation of activated T cells. CM/HIV+ showed reduced CD4 T lymphocytes. **D** Heatmap of CSF parameters. The mean of each parameter was calculated, scaled, centered and clustered hierarchically. Abbreviations - AUC: area under the curve, Bc: B lymphocytes, BCBD: blood-CSF-barrier disruption, brightNK: CD56^bright^ natural killer cells, CM: cryptococcal meningitis, CD45: leukocytes, cMono: classical monocytes, CSF: cerebrospinal fluid, Ctrl: healthy control group, dimNK: CD56^dim^ natural killer cells, Granulo: proportion of granulocytes, HIV: human immunodeficiency virus positive, immunocompromised control group, HLA-DR Tc: activated T cells, IgA: immunoglobulin A,  IgG: immunoglobulin G,  IgM: immunoglobulin M, Lympho: proportion of lymphocytes,   iMono: intermediate monocytes, mono: monocytes, ncMono: non-classical monocytes, NK: natural killer cells, NKT: natural killer T cells, OCB: oligoclonal bands, PCA: principal component analysis, PC: principal component, plasma: plasma cells, ROC: receiver operating characteristic analysis, Tc: T lymphocytes

​​We next focused on changes in cellular subpopulations in blood and CSF of patients with CM by capitalizing on available multicolor flow cytometry (mFC) data at our center. We integrated standard CSF parameters and mFC data of CSF and performed principle component analysis (PCA). CM/HIV- patients revealed broad clustering, indicating high heterogeneity within the group. The Ctrl group showed overlap with CM/HIV- patients. CM/HIV+ patients and the HIV control group were clustered more densely and distinctly separated from the other groups (Fig. 2A). Principal components identified the parameters differentiating individual groups (Fig. 2B).

Increased IgG ratio and CSF leukocytes (CD45 +) and decreased CSF CD4 positive T cells were the main drivers that separated HIV from Ctrl and indicated HIV infection. Increased CSF protein, albumin and IgA ratio, and granulocytes mainly drove the differences between CM and HIV as well as CM and Ctrl and indicated cryptococcal meningitis (Fig. 2B).

In the next step, we compared the individual CSF cell populations between the three groups (Fig. 2C, Supplementary Fig. 1). CM/HIV- patients showed a significant increase of NK cells with a dominance of bright NK cells and increase of NKT cells in the CSF (Fig. 2C), next to the previously mentioned increase of CSF protein, lactate, albumin and immunoglobulin ratios (Fig. 1). NK cells have been reported to play a decisive role in the early immune response to fungal infection. Our findings thus indicate an activation of innate immune mechanisms in the CSF compartment and underline the importance of NK cells in the defense of CM. Both CM/HIV+ and CM/HIV- patients revealed an increase of HLA-DR CD4 T cells, hinting to activation of the adaptive immune system too (Fig. 2C).

Heatmaps revealed partial overlap of CSF parameters in the CM/HIV+ and HIV control group, including decrease of CD4 positive T cells and CD4/CD8 ratio, as expected due to HIV infection. Further, heatmaps also displayed a higher increase of NK cells and granulocytes in CM/HIV- compared to CM/HIV+ patients (Fig. 2D). Intrathecal immunoglobulins showed higher increase in CM/HIV+ patients compared to the CM/HIV- group (Fig. 2D). We next quantified the ability of individual parameters in distinguishing groups by calculating the area under the curve (AUC) in receiver operating curve (ROC) analysis. This returned lactate level in CSF as the best parameter (AUC 0.9102) when comparing CM and HIV, followed by glucose (AUC 0.8769) and NK cells (AUC 0.8241) (Supplementary Fig. 4, Supplementary Table 2). Further, ROC analysis identified immunoglobulin G level in CSF (AUC 0. 9722) as the best parameter to distinguish CM from Ctrl, followed by albumin ratio, immunoglobulin ratios over all subclasses, protein level in the CSF and BCBD (AUC ≥ 0.9) (Supplementary Fig. 4, Supplementary Table 2). The differentiation of HIV patients to Ctrl was mainly determined by diminished CD4/CD8 ratio and the presence of OCBs as illustrated in heatmaps (Fig. 2D). ROC analysis identified immunoglobulin A, albumin and protein levels in the CSF when comparing HIV to Ctrl (AUC ≥ 0.9) (Supplementary Fig. 4A, Supplementary Table 2).

### CD56^bright^ NK and NKT cells increase in the blood in CM/HIV-

**Fig. 3:**
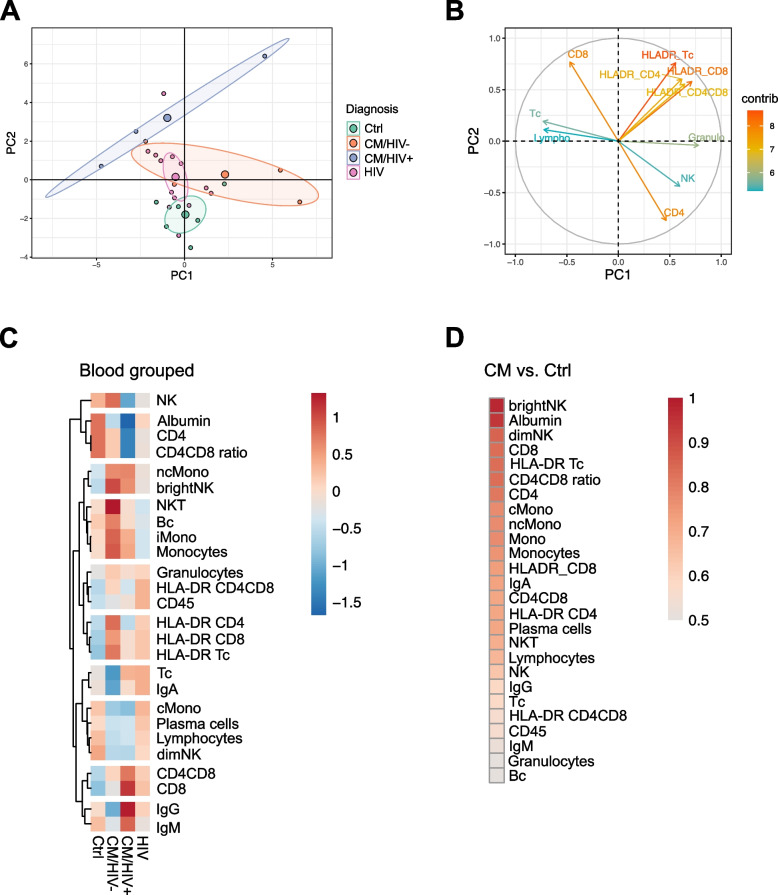
Flow cytometry analysis of blood. Blood parameters distinguish CM from control groups partially. **A** Principal component analysis of blood revealed high heterogeneity within CM patients and partial overlap with HIV patients. Each patient is illustrated as a multidimensional data point. Large circles represent the means of each group (CM/HIV+ blue, CM/HIV- orange, HIV pink, Ctrl green). Ellipses of the same color represent the confidence intervals of each group. **B** Principal components (PCs) comprise multidimensional parameters.  Parameters with the highest distinctive power determine PC1 and PC2 and are represented by the length of their vector. **C **Heatmap of blood parameters. The mean of each parameter was calculated, scaled, centered and clustered hierarchically. **D** Receiver operating characteristic (ROC) analysis of blood parameters. Sensitivity and specificity are represented by area under the curve (AUC) values, which range from 1 (perfect distinction) to 0.5 (uninformative). Heatmaps illustrate the hierarchy of all parameters according to their distinctive power. When comparing blood parameters, CD56bright NK cells (AUC = 0.9795) were the best parameter to distinguish CM from Ctrl, followed by albumin (AUC = 0.9444). Abbreviations - abs: cell counts/ µl, AUC: area under the curve, Bc: B lymphocytes, BCBD: blood-CSF-barrier disruption, brightNK: CD56^bright^ natural killer cells, CM: cryptococcal meningitis, cMono: classical monocytes, CSF: cerebrospinal fluid, Ctrl: healthy control group, dimNK: CD56^dim^ natural killer cells, Granulo: granulocytes, HIV: human immunodeficiency virus positive, immunocompromised control group, HLA-DR Tc: activated T cells, IgA: immunoglobulin A,  IgG: immunoglobulin G,  IgM: immunoglobulin M,  iMono: intermediate monocytes, Lympho: lymphocytes, mono: monocytes, ncMono: non-classical monocytes, NK: natural killer cells, NKT: natural killer T cells, OCB: oligoclonal bands, PCA: principal component analysis, PC: principal component, plasma: plasma cells, ROC: receiver operating characteristic analysis, Tc: T lymphocytes

We next analyzed flow cytometry data of blood samples collected in parallel to CSF. Similar to our observations in the CSF, PCA showed high heterogeneity of CM groups, whereas the HIV and Ctrl groups were more homogenous. Ctrl were separable from the CM group and the HIV group. The CM/HIV+ and CM/HIV- patients were separated clearly whereas the CM/HIV- group showed overlap within the HIV control group (Fig. 3A). Increase of CD8 positive T cells and activated T cell populations as well as decrease of NK cells and CD4 positive T cells were the main drivers of the PCA separation (Fig. 3B). Both CM/HIV+ and HIV patients showed an increase of T cells, whereas CM/HIV- patients revealed a strong increase in NK and NKT cells and increase of B lymphocytes in the blood as illustrated by heatmaps (Fig. 3C) and individual plots (Supplementary Fig. 2). Decreased albumin and classical monocytes were identified as the most distinct parameters in heatmaps when comparing blood of CM groups and HIV (Fig. 3C). Assessed by ROC analysis, CD56^bright^ NK cells (AUC 0.9795) were the parameter with the highest distinctive power when comparing CM to healthy controls (Fig. 3D). ROC analysis identified albumin (AUC 0.8141), NKT cells, monocyte counts and classical monocytes (AUC 0.8131) as the most distinct blood parameters when comparing CM to HIV patients (Supplementary Fig. 4B, Supplementary Table 2). This indicates that blood could serve as a disease surrogate in immunocompromised patients.

### Slow reconstitution kinetics of CSF abnormalities during antifungal therapy

**Fig. 4:**
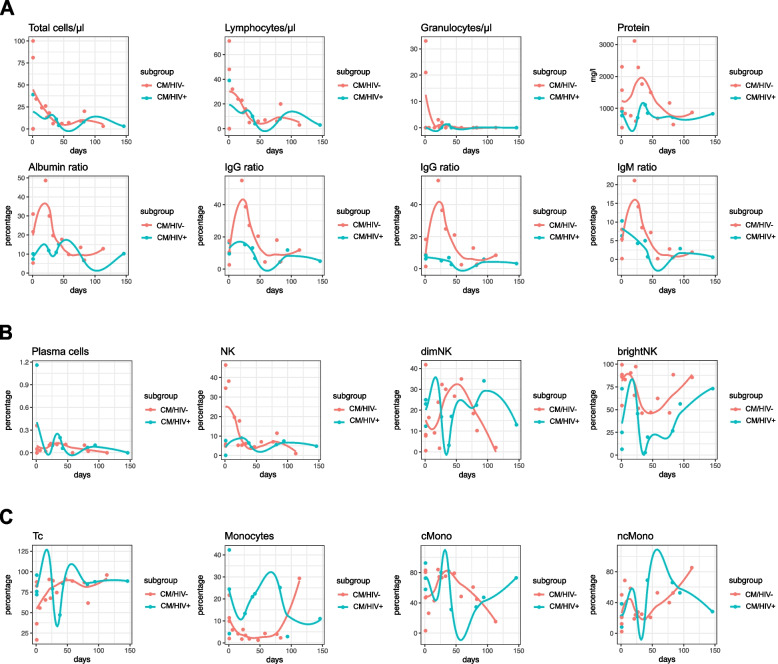
Reconstituition kinetics of CSF parameters under antifungal therapy. Slow normalization of standard CSF parameters and variable courses of NK cell populations after induction of antifungal therapy. Parameters over the course of time of the CM/HIV+ group are represented as lines in green. Lines of the CM/HIV- group are represented in red color. Flow cytometry of CSF was performed at the first presentation of each patient and during follow-up in different intervals. Antifungal therapy was started within days after the first diagnosis of CM. Samples were taken in different time intervals, trends of each parameter are represented over a period of 150 days. First sample (day 0) of two patients were taken during follow up, both were already on antifungal therapy (Patient 10: 10 months after first diagnosis; Patient 24: 30 months after first diagnosis). Correlation analysis was performed with Pearson’s correlation coefficient and p values were adjusted with the Benjamini–Hochberg method. **A** Reconstitution kinetics of cells, protein and immunoglobulins in CSF. **B **Reconstitution kinetics of plasma cells, NK cells and NK subtypes. **C** Reconstitution kinetics of T cell, monocytes and monocyte subpopulations. Abbreviations - Bc: B lymphocytes, BCBD: blood-CSF-barrier disruption, brightNK: CD56^bright^ natural killer cells, CM: cryptococcal meningitis, cMono: classical monocytes, CSF: cerebrospinal fluid, Ctrl: healthy control group, dimNK: CD56^dim^ natural killer cells, Granulo: proportion of granulocytes, HIV: human immunodeficiency virus positive, immunocompromised control group, HLA-DR Tc: activated T cells, IgA: immunoglobulin A,  IgG: immunoglobulin G,  IgM: immunoglobulin M,  iMono: intermediate monocytes, Lympho: proportion of lymphocytes, mono: monocytes, ncMono: non-classical monocytes, NK: natural killer cells, NKT: natural killer T cells, OCB: oligoclonal bands, Plasma: plasma cells, ROC: receiver operating characteristic analysis, Tc: T lymphocytes

We were interested in how fungal-associated CSF abnormalities reconstituted under treatment. Antifungal therapy was induced in all patients with diagnosis. CM patients received further CSF analyses during follow-up examinations in different time intervals (5 days up to 11 years after first LP). To identify potential biomarker candidates of a therapy response, we visualized all parameters over 150 days after the first LP (Fig. 4, Supplementary Fig. 3) and compared CM/HIV+ to CM/HIV- patients.

Increased cell count, protein and intrathecal immunoglobulins in CSF decreased within a few days after induction of therapy (Fig. 4A). Initial protein levels were slightly higher in CM/HIV- compared to CM/HIV+ patients but decreased to normal protein levels after approximately 75 days in both groups. One hundred days after the first LP, CSF perturbations were mostly reversed. Percentages of plasma cells, NK cells and granulocytes were decreasing over time (Fig. 4B, Supplementary Fig. 3). Initial proportions of NK cells and CD56^bright^ NK cells were higher in the CM/HIV- group whereas CD56^dim^ NK cells were higher in the CM/HIV+ group. CD56^bright^ NK cells rapidly decreased after induction of antifungal therapy and reached the lowest level by day 40 to 50, followed by a slow increase afterwards in both CM/HIV+ and CM/HIV- patients (Fig. 4B). As expected in healthy patients, plasma cells were not detected in most patients after 50 to 150 days (Fig. 4B). T cells displayed a slow increase in CM/HIV- patients over time (Fig. 4C). The proportions of monocytes and monocyte subsets exhibited variable kinetics in the CM/HIV+ and CM/HIV- group (Fig. 4C). Of note, this suggests a *relative* increase of the previously reduced cell proportion (Fig. 4B, 4C, Supplementary Fig. 3).

## Discussion

*Cryptococcus neoformans* can cause fatal meningitis and meningoencephalitis in immunocompromised individuals [1, 3]. Fungal infection with *Cryptococcus neoformans* has been studied with focus on virulence factors and survival mechanisms of the pathogen [2, 3, 6, 14–16], though the immunological response in the human organism has been poorly understood so far. We present a retrospective study of flow cytometry data of CSF and blood in patients with CM and compare them to immunocompromised HIV positive patients without CM and a group of HIV negative healthy controls.

Our study was limited due to its retrospective design and a rather small cohort of patients with CM. We screened our clinical database and selected patients with CM and available multicolor flow cytometry data. As flow cytometry is only performed during regular working hours from 8 a.m. to 4 p.m., mFC data are limited. Due to partition of three HIV patients in the CM group, overlapping features with the immunocompromised HIV positive control group could be observed. Though elevated intracranial pressure commonly occurs with CM [1, 4, 5], CSF opening pressure was not assessed or documented in our clinical data system, so no information could be provided regarding this aspect.

Changes of standard CSF parameters associated with fungal meningitis have been reported in the literature before and we could reproduce those findings including decrease of glucose and increase of lactate, elevation of protein and immunoglobulins and the presence of severe blood-CSF-barrier disruption [1, 6]. Those changes have been reported in infectious diseases of the central nervous system before, though they are not specific for fungal pathogens and have been noticed in bacterial meningitis/ meningoencephalitis too [17, 18].

The cellularity of CSF in CM has rarely been investigated before. Due to their size and capsular phenotype, cryptococcal pathogens are often confounded with lymphocytes in microscopy of blood or CSF. By performance of flow cytometry of CSF, we aimed to identify cellular subpopulations that are involved in CNS invasion and compartment-specific immune response and might indicate more specifically cryptococcal infection. Flow cytometry analysis in CM has been reported before [11, 12], but reports have been lacking comparison to immunocompromised patients without CM and healthy controls.

Scriven et al*.* [8] previously presented a study detecting *Cryptococcus* in CSF by flow cytometry. In this work, gating along sideward scatter (SSC) and CD45 allowed detection of a cell population that was expected to indicate the presence of cryptococcal pathogens in the CSF (CD45 negative population). CD45 is used as a panleukocyte marker. When gating along FSC/SSC, confounding of *Cryptococci* and lymphocytes is possible, but our gating strategy followed selection of CD45 positive cells in the second step to further categorize the presence of lymphocytes, monocytes and granulocytes. CD45 is not expressed on cryptococcal pathogens. Thus, confounding of lymphocytes and *Cryptococcus* is unlikely in our flow cytometry analysis. Moreover, we displayed changes of cellular subpopulations over 150 days after induction of antifungal therapy which has not been investigated before.

First, routine CSF analysis revealed severe blood-CSF-barrier disruption and positive oligoclonal bands in patients with CM. Intrathecal immunoglobulin synthesis was higher in CM/HIV+ , compared to CM patients with other immunocompromised conditions (CM/HIV-). Flow cytometry analysis further revealed an increase of plasma cells and monocytes with a dominance of classical monocytes in the CSF of the CM/HIV+ group. Changes in the microvascular environment and BCBD have been reported in HIV patients before and have been attributed to HIV infection affecting the CNS [19–21].

In CM/HIV- patients, NK cells with a dominance of CD56^bright^ NK cells and NKT cells were highly increased in CSF and blood, pointing to activation of the innate immune system in both compartments in response to dissemination and CNS invasion of *Cryptococcus neoformans*. Both NK cells and NKT cells have been shown to play an important role in the early stages of antifungal immune response. NK cells perform various defense mechanisms to control and eliminate fungal pathogens, including direct toxicity via cytotoxic molecules, but also death-receptor mediated apoptosis and antibody-dependent cell-mediated cytotoxicity [22–25]. NK cells induce the synthesis of Type I mediators and interact with other cells of the innate and adaptive immune system, creating a link in between innate and adaptive immune mechanisms that contribute to the elimination of the fungus [23].

Both CM/HIV- as well as CM/HIV+ patients displayed elevation of activated CD4 positive and activated CD4CD8 positive T cells in CSF. The activation of T lymphocytes in patients with CM implicates further involvement of adaptive immune mechanisms in the CNS as a response to fungal infection. It has been shown that NKT cells promote a T_H_1-dominating T cell response in the early phase of systemic infection by *Cryptococcus neoformans* [24]. Thus, they contribute to an immediate immune response but also to continuous protective immunity.

The importance of NK cells in infectious disease is not limited to fungal infection though. NK cells have also been shown to be involved in viral and bacterial infections e.g. with cytomegalovirus or Epstein-Barr-Virus, Escherichia coli or Listeria monocytogenes [23]. An increase of NK cells in the CSF has been observed in viral and bacterial meningitis too [18] though direct comparison of cellular subsets in the CSF of viral vs. bacterial vs. fungal meningitis has not been reported before.

Changes of cellular subsets in the CSF in CM after induction of antifungal therapy have been reported over a period of two weeks and focused on monocyte and NK subsets [26]. Our retrospective study displays trends of various cell subpopulations in the CSF over 150 days after first lumbar puncture.

Next to normalization of protein, immunoglobulin ratios as well as glucose and lactate, we observed slow reconstitution of immune cell alterations after induction of antifungal therapy, especially in plasma cells, granulocytes and NK cells.

After induction of antifungal therapy, the CM/HIV- group initially displayed an increase of B cells, bright NK cells and activated T lymphocytes, whereas NK and NKT cells rapidly decreased. After reaching a peak at about day 10, activated T cells slowly decreased, followed by a second increase of NK, NKT and T cells after day 40. These variable courses hint to involvement of innate and adaptive immune mechanisms in the CNS in the course of fungal infection and elimination. CM/HIV+ patients initially displayed an increase of NK and bright NK cells, but a decrease of activated T lymphocytes. This hints to a weaker adaptive immune response and greater importance of the innate immune response in this group.

Our study provides a hypothesis for cellular interactions in CM and to predict immunological mechanisms in the CSF in response to fungal CNS infection. Further investigations are needed to analyze the involvement and interactions of immune cells as well as intracellular operations and to deeply understand immune cell mechanisms in response to CM.

In this study, we aimed to investigate the host cellular response in the cerebrospinal fluid of patients with CM. The study was limited regarding its retrospective design and limited availability of multicolor flow cytometry data, resulting in a rather small cohort of CM patients.

In summary, we represent flow cytometry analysis of CSF in a group of patients with CM in comparison to immunocompromised HIV patients without meningitis and healthy controls. We detected a strong increase of NK cells in the CSF in CM, indicating activation of an innate immune response in the CSF compartment. We revealed slow reconstitution of standard CSF parameters and heterogenous trends of CSF cell subsets after initiation of antifungal therapy. Performance of flow cytometry on CSF indicates involvement of innate and adaptive immune responses to CM and illustrates slow reconstitution of immune cell alterations after induction of antifungal therapy in follow-up examinations.

## Supplementary Information

Supplementary Material 1.

Supplementary Material 2.

Supplementary Material 3.

Supplementary Material 4.

Supplementary Material 5.

Supplementary Material 6.

## Data Availability

Data are available upon request to the corresponding author.

## Abbreviations

AIDS: Acquired Immunodeficiency Syndrome
AUC: Area Under the Curve
BCBD: Blood-CSF-barrier disruption
CM: Cryptococcal meningitis
cMono: Classical monocytes
CNS: Central nervous system
Cr-Ag: Cryptococcal antigen
CSF: Cerebrospinal fluid
Ctrl: Healthy controls
FSC: Forward scatter channel
Granulo: Granulocytes
HIV: Human immunodeficiency virus
IgA: Immunoglobulin A
IgG: Immunoglobulin G
IgM: Immunoglobulin M
iMono: Intermediate monocytes
Lympho: Lymphocytes
LP: Lumbar punctuare
mFC: Multicolor flow cytometry data
Mono: Monocytes
ncMono: Non-classical monocytes
NK: Natural killer cells
NKT: Natural killer T cells
OCB: Oligoclonal bands
PC: Principal component
PCA: Principal Component Analysis
ROC: Receiver Operating Characteristic analysis
SSC: Sideward scatter channel

## References

[1] Fisher KM, et al. Cryptococcal meningitis: a review for emergency clinicians. Intern Emerg Med. 2021;16(4):1031–42. 33420904 10.1007/s11739-020-02619-2

[2] Liu TB, Perlin DS, Xue C. Molecular mechanisms of cryptococcal meningitis. Virulence. 2012;3(2):173–81. 22460646 10.4161/viru.18685 PMC3396696

[3] Zaragoza O. Basic principles of the virulence of Cryptococcus. Virulence. 2019;10(1):490–501. 31119976 10.1080/21505594.2019.1614383 PMC6550552

[4] Robertson EJ, et al. Cryptococcus neoformans ex vivo capsule size is associated with intracranial pressure and host immune response in HIV-associated cryptococcal meningitis. J Infect Dis. 2014;209(1):74–82. 23945372 10.1093/infdis/jit435 PMC3864387

[5] Alanazi AH, Adil MS, Lin X, Chastain DB, Henao-Martínez AF, Franco-Paredes C, Somanath PR. Elevated intracranial pressure in cryptococcal meningoencephalitis: examining old, new, and promising drug therapies. Pathogens. 2022;11(7):783. 10.3390/pathogens11070783. 10.3390/pathogens11070783 PMC9321092 35890028

[6] Colombo AC, Rodrigues ML. Fungal colonization of the brain: anatomopathological aspects of neurological cryptococcosis. An Acad Bras Cienc. 2015;87(2 Suppl):1293–309. 26247147 10.1590/0001-3765201520140704

[7] Scriven JE, et al. The CSF Immune Response in HIV-1-Associated Cryptococcal Meningitis: Macrophage Activation, Correlates of Disease Severity, and Effect of Antiretroviral Therapy. J Acquir Immune Defic Syndr. 2017;75(3):299–307. 28346317 10.1097/QAI.0000000000001382 PMC5469563

[8] Scriven JE, et al. Flow Cytometry To Assess Cerebrospinal Fluid Fungal Burden in Cryptococcal Meningitis. J Clin Microbiol. 2016;54(3):802–4. 26719441 10.1128/JCM.03002-15 PMC4767993

[9] Heming M, et al. Immune Cell Profiling of the Cerebrospinal Fluid Provides Pathogenetic Insights Into Inflammatory Neuropathies. Front Immunol. 2019;10:515. 30984164 10.3389/fimmu.2019.00515 PMC6448021

[10] Heming M, et al. Supporting the differential diagnosis of connective tissue diseases with neurological involvement by blood and cerebrospinal fluid flow cytometry. J Neuroinflammation. 2023;20(1):46. 36823602 10.1186/s12974-023-02733-w PMC9951507

[11] Peterson RA, Cavanaugh JE. Ordered quantile normalization: a semiparametric transformation built for the cross-validation era. J Appl Stat. 2020;47(13–15):2312–27. 35707424 10.1080/02664763.2019.1630372 PMC9042069

[12] Lê S, Josse J, Husson F. FactoMineR: An R Package for Multivariate Analysis. J Stat Softw. 2008;25(1):1–18.

[13] Robin X, et al. pROC: an open-source package for R and S+ to analyze and compare ROC curves. BMC Bioinformatics. 2011;12:77. 21414208 10.1186/1471-2105-12-77 PMC3068975

[14] Casadevall A, et al. The capsule of Cryptococcus neoformans. Virulence. 2019;10(1):822–31. 29436899 10.1080/21505594.2018.1431087 PMC6779390

[15] Coelho C, Bocca AL, Casadevall A. The tools for virulence of Cryptococcus neoformans. Adv Appl Microbiol. 2014;87:1–41. 24581388 10.1016/B978-0-12-800261-2.00001-3

[16] Maziarz EK, P.J. Cryptococcosis. Infect Dis Clin North Am. 2016;30:179–206. 26897067 10.1016/j.idc.2015.10.006 PMC5808417

[17] Hasbun R. Progress and Challenges in Bacterial Meningitis: A Review. JAMA. 2022;328(21):2147–54. 36472590 10.1001/jama.2022.20521

[18] Lepennetier G, et al. Cytokine and immune cell profiling in the cerebrospinal fluid of patients with neuro-inflammatory diseases. J Neuroinflammation. 2019;16(1):219. 31727097 10.1186/s12974-019-1601-6 PMC6857241

[19] Toborek M, et al. Mechanisms of the blood-brain barrier disruption in HIV-1 infection. Cell Mol Neurobiol. 2005;25(1):181–99. 15962513 10.1007/s10571-004-1383-x PMC11529531

[20] Sun Y, et al. Disruption of blood-brain barrier: effects of HIV Tat on brain microvascular endothelial cells and tight junction proteins. J Neurovirol. 2023;29(6):658–68. 37899420 10.1007/s13365-023-01179-3

[21] Rahimy E, et al. Blood-Brain Barrier Disruption Is Initiated During Primary HIV Infection and Not Rapidly Altered by Antiretroviral Therapy. J Infect Dis. 2017;215(7):1132–40. 28368497 10.1093/infdis/jix013 PMC5426376

[22] Vivier E, et al. Functions of natural killer cells. Nat Immunol. 2008;9(5):503–10. 18425107 10.1038/ni1582

[23] Schmidt S, Tramsen L, Lehrnbecher T. Natural Killer Cells in Antifungal Immunity. Front Immunol. 2017;8:1623 29213274 10.3389/fimmu.2017.01623 PMC5702641

[24] Galvez, N.M.S, et al. Type I Natural Killer T Cells as Key Regulators of the Immune Response to Infectious Diseases. Clin Microbiol Rev,. 2021;34(2):e00232. 33361143 10.1128/CMR.00232-20 PMC7950362

[25] Cooper M.A, Fehniger T.A, Caligiuri M.A. The biology of human natural killer-cell subsets. Trends Immunol. 2001;22(11):633–40. 11698225 10.1016/s1471-4906(01)02060-9

[26] Meya DB, et al. Cellular immune activation in cerebrospinal fluid from ugandans with cryptococcal meningitis and immune reconstitution inflammatory syndrome. J Infect Dis. 2015;211(10):1597–606. 25492918 10.1093/infdis/jiu664 PMC4407762

