# Supplementary material for: Severe CSF immune cell alterations in cryptococcal meningitis gradually resolve during antifungal therapy
Source: BMC Neurol. 2024 Jul 3;24:229. doi: 10.1186/s12883-024-03742-9 (PMC11221170; doi:10.1186/s12883-024-03742-9)
Supplement: Supplementary file 3 — Supplementary Material 3. [file 12883_2024_3742_MOESM3_ESM.pdf]

Supplementary Figure 1: Comparison of cellular subpopulations in the CSF of CM and control groups

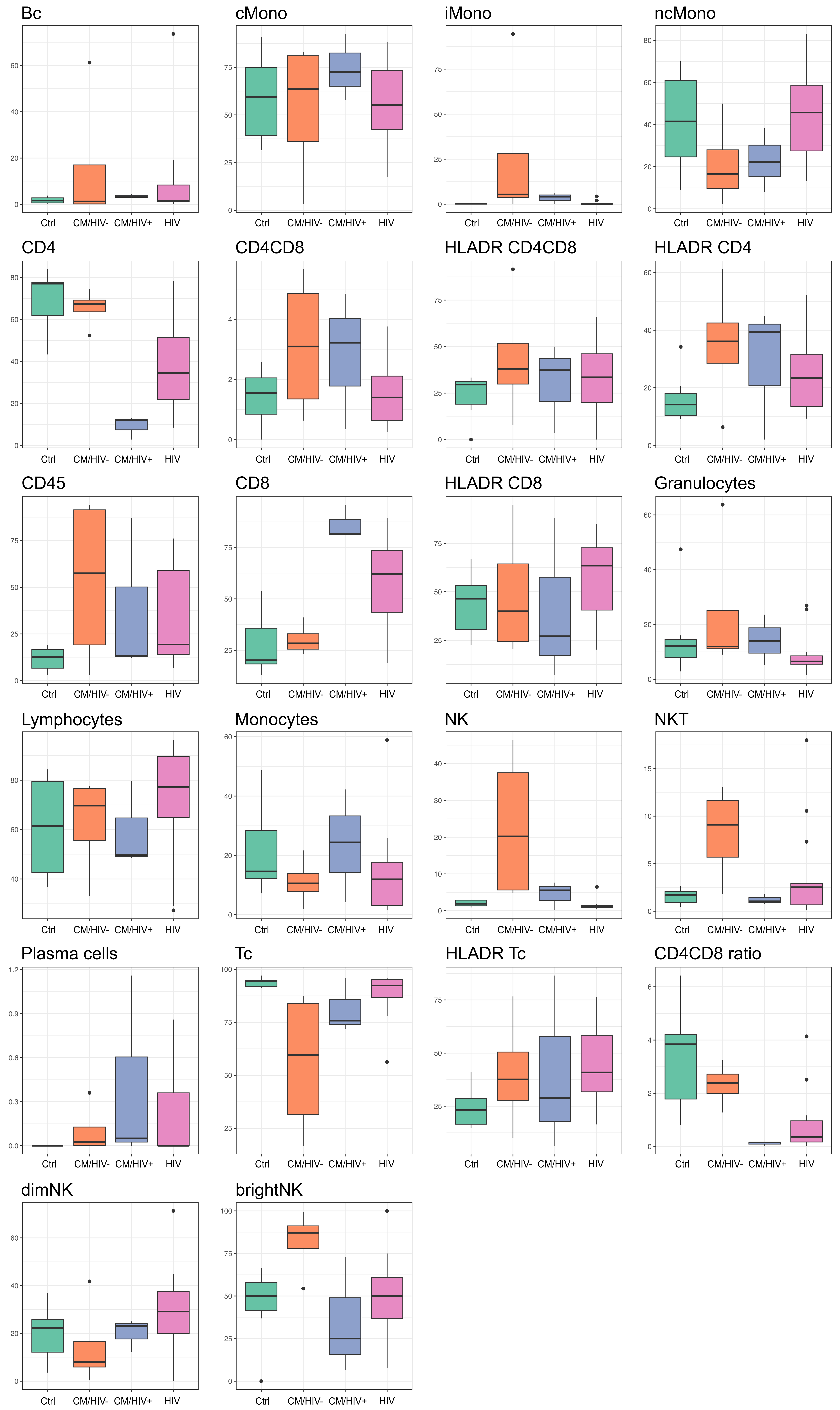

Supplementary Figure 1: Plotting of individual mFc parameters revealed a significant increase of NK cells in the CSF of the CM group. Other cellular subpopulations showed no alteration in the CSF of CM patients compared to HIV and Ctrl. Abbreviations - Bc: B lymphocytes, brightNK: CD56bright natural killer cells, CM: cryptococcal meningitis, CD45: leukocytes, cMono: classical monocytes, CSF: cerebrospinal fluid, Ctrl: healthy control group, dimNK: CD56dim natural killer cells, HIV: human immunodeficiency virus positive, immunocompromised control group, HLA-DR Tc: activated T cells, iMono: intermediate monocytes, mFC: multicolor flow cytometry data, ncMono: non-classical monocytes, NK: natural killer cells, NKT: natural killer T cells, Tc: T lymphocytes
