# Supplementary material for: Severe CSF immune cell alterations in cryptococcal meningitis gradually resolve during antifungal therapy
Source: BMC Neurol. 2024 Jul 3;24:229. doi: 10.1186/s12883-024-03742-9 (PMC11221170; doi:10.1186/s12883-024-03742-9)
Supplement: Supplementary file 5 — Supplementary Material 5. [file 12883_2024_3742_MOESM5_ESM.pdf]

Supplementary Figure 3: Reconstitution kinetics of CSF parameters after induction of antifungal therapy over a period of 150 days

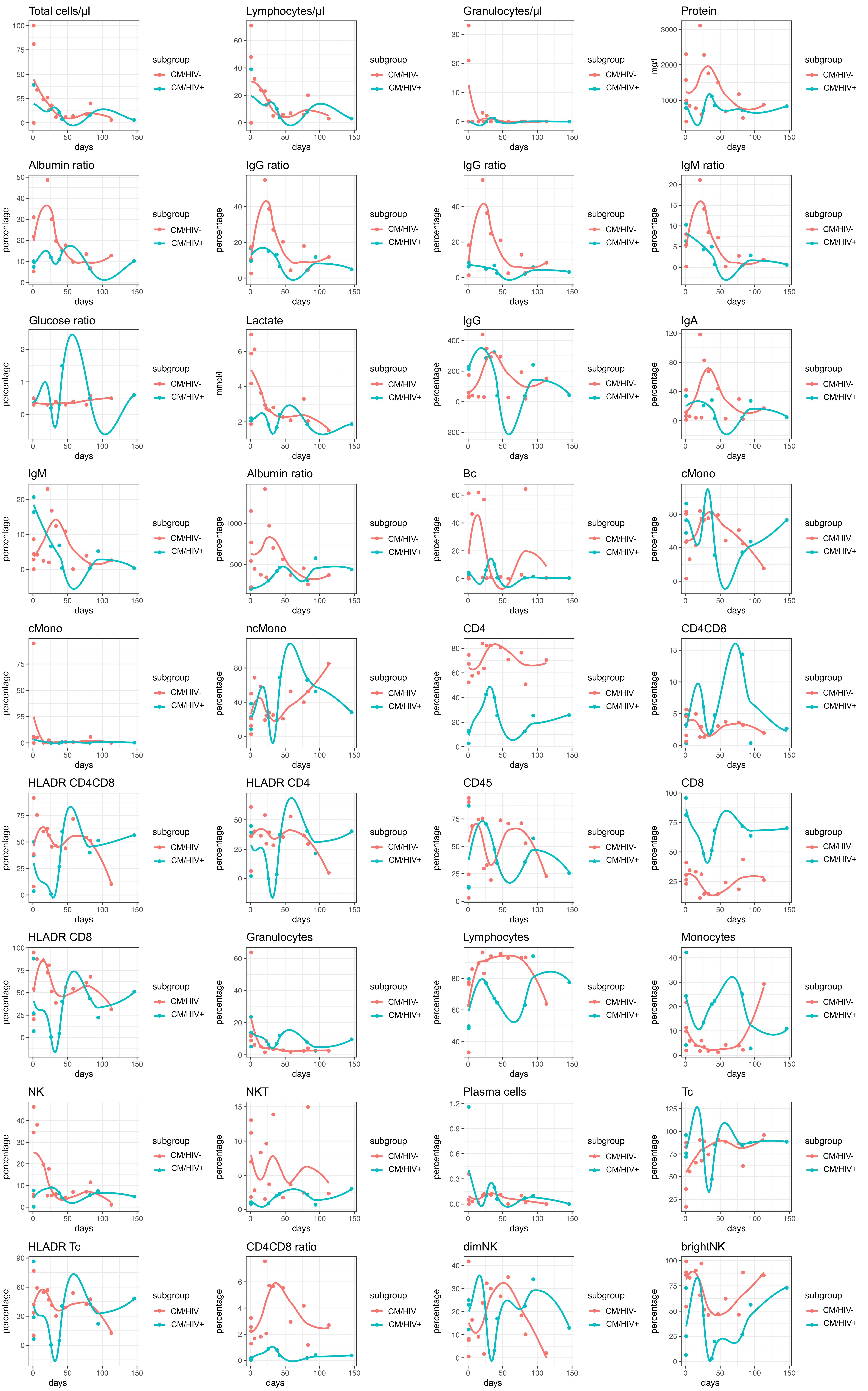

Supplementary Figure 3: Slow normalization of standard CSF parameters and NK cells after induction of antifungal therapy. Parameters of patients with cryptococcal meningitis are presented as individual lines in red for CM/HIV- patients and in green for CM/HIV+ patients. Flow cytometry of CSF was performed at the first presentation of each patient and during follow-up in different intervals. Antifungal therapy was started within days after the first diagnosis of cryptococcal meningitis. Samples were taken in different time intervals, trends of each parameter are represented over a period of 150 days. First sample (day 0) of two patients were taken during follow up, both were already on antifungal therapy (Patient 10: 10 months after first diagnosis; Patient 24: 30 months after first diagnosis). Correlation analysis was performed with Pearson's correlation coefficient and p values were adjusted with the Benjamini-Hochberg method.

Abbreviations - Bc: B lymphocytes, BCBD: blood-CSF-barrier disruption, brightNK: CD56bright natural killer cells, CM: cryptococcal meningitis, cMono: classical monocytes, CSF: cerebrospinal fluid, Ctrl: healthy control group, dimNK: CD56dim natural killer cells, HIV: human immunodeficiency virus positive, immunocompromised control group, HLADR Tc: activated T cells, IgA: immunoglobulin A, IgG: immunoglobulin G, IgM: immunoglobulin M, iMono: intermediate monocytes, ncMono: non-classical monocytes, NK: natural killer cells, NKT: natural killer T cells, OCB: oligoclonal bands, ROC: receiver operating characteristic analysis, Tc: T lymphocytes
