# Supplementary material for: Severe CSF immune cell alterations in cryptococcal meningitis gradually resolve during antifungal therapy
Source: BMC Neurol. 2024 Jul 3;24:229. doi: 10.1186/s12883-024-03742-9 (PMC11221170; doi:10.1186/s12883-024-03742-9)
Supplement: Supplementary file 6 — Supplementary Material 6. [file 12883_2024_3742_MOESM6_ESM.pdf]

Supplementary Figure 4: ROC analysis of CSF and blood parameters

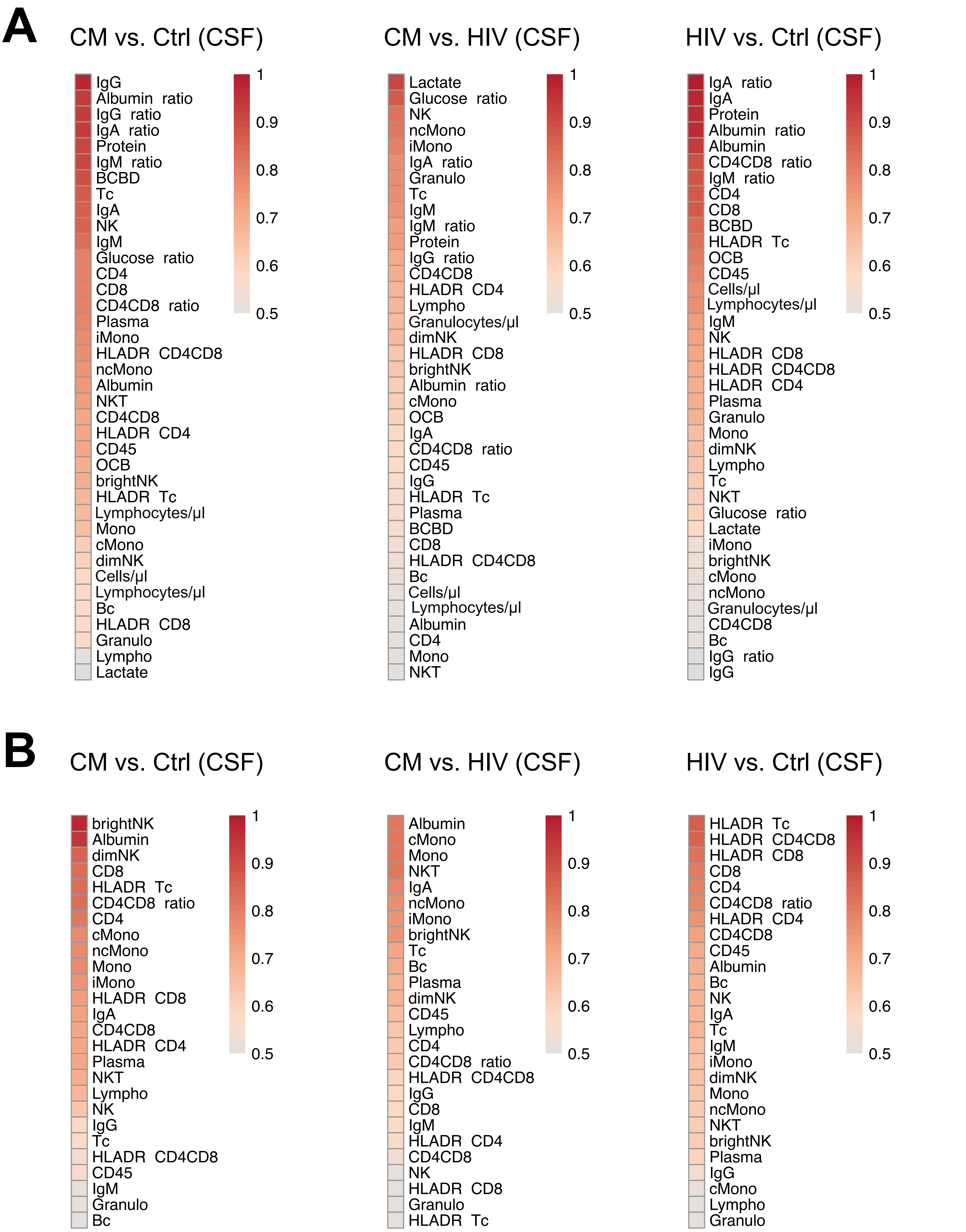

Supplementary Figure 4: Receiver operating characteristic (ROC) analysis of CSF parameters. Sensitivity and specificity are represented by area under the curve (AUC) values, which range from 1 (perfect distinction) to 0.5 (uninformative). Heatmaps illustrate the hierarchy of all parameters according to their distinctive power when comparing CSF (A) and blood parameters (B) of CM vs. Ctrl, CM vs. HIV and HIV vs. Ctrl. AUC values are listed in Supplementary Table 2.

Abbreviations – abs: absolute cell counts, Bc: B lymphocytes, BCBD: blood-CSF-barrier disruption, brightNK: CD56bright natural killer cells, CM: cryptococcal meningitis, cMono: classical monocytes, CD45: leukocytes, CSF: cerebrospinal fluid, Ctrl: healthy control group, dimNK: CD56dim natural killer cells, Granulo: granulocytes, HIV: human immunodeficiency virus positive (immunocompromised control group), HLADR Tc: activated T cells, IgA: immunoglobulin A, IgG: immunoglobulin G, IgM: immunoglobulin M, iMono: intermediate monocytes, Lympho: lymphocytes, mFC: multicolor flow cytometry data, Mono: monocytes, ncMono: non-classical monocytes, NK: natural killer cells, NKT: natural killer T cells, OCB: oligoclonal bands, Plasma: plasma cells, Tc: T lymphocytes
